# PReS-FINAL-2126: How to follow up children with Raynaud syndrome - recommendations based on the Hamburg consensus meeting 2012

**DOI:** 10.1186/1546-0096-11-S2-P138

**Published:** 2013-12-05

**Authors:** T Constantin, C Pain, N Toplak, M Moll, I Konert, DP Piotto, NA Ayaz, D Nemkova, P Hoeger, M Cutolo, V Smith, I Foeldvari, Juvenile Scleroderma Working Group

**Affiliations:** 1 Semmelweis University, Budapest, Hungary; 2 Alder Hey Childrens Hospital, Liverpool, UK; 3 University of Ljubljana, Ljubljana, Slovenia; 4 University of Tuebingen, Tuebingen, Germany; 5 Universittsklinikum Hamburg-Eppendorf, Hamburg, Germany; 6 Universidade Federal de So Paulo, Sao Paolo, Brazil; 7 Sultan Sleyman Education and Research Hospital, Istanbul, Turkey; 8 University of Prague, Prague, Czech Republic; 9 Wilhelmstift Hospital for Sick Children, Hamburg, Germany; 10 University Medical School of Genova, Genova, Italy; 11 Gent University Hospital, Gent, Belgium; 12 Hamburger Zentrum fr Kinder- und Jugendrheumatologie, Hamburg, Germany

## Introduction

Raynauds phenomenon (RP) can be the first symptom of a connective tissue disease in children, in particular juvenile systemic scleroderma (JSSC) or systemic lupus erythematosis (SLE). However, the prevalence of RP in healthy school children has been shown to be as high as 15%[1]. There are currently no guidelines or agreed management strategies amongst Paediatric Rheumatologists on how to differentiate primary from secondary RP or how often patients require evaluation.

## Objectives

To develop consensus standards for good clinical practice for children with RP.

## Methods

A consensus meeting was organized in the frame of the PRES scleroderma working group. A nominal group technique was used. 75% consensus was defined as agreement.

## Results

1. All patients with RP should be screened with an ANA test.

2. All ANA positive patients should be screened for scleroderma-specific antibodies (e.g. anti-SCL 70 and anti-centromere antibodies).

3. All patients with RP should be investigated by capillaroscopy. Capillaroscopy will be classified into "normal", "aspecific changes" or "scleroderma pattern".

4. All patients who have additional symptoms pointing to a definite connective tissue disease should be evaluated according to disease specific guidelines.

5. ANA-negative and capillaroscopy-negative patients should be followed-up at least every 6 months.

6. ANA positive patients without disease-specific antibodies and with negative capillaroscopy findings should be followed-up at least every 6 months.

7. ANA and disease-specific antibody positive patients should have organ specific evaluation according to symptoms, examination and relevant to that particular disease e.g. patients who are ANA and Scl-70 positive may need organ specific evaluation for JSSC as per the Juvenile systemic sclerosis inception cohort protocol (http://www.juvenile-scleroderma.com).

8. ANA-positive patients, who have no disease specific antibody but have positive capillaroscopy results, should be followed-up at least every 3 months.

9. ANA-negative patients with positive capillaroscopy result should be followed-up at least every 6 months.

10. The group could not reach an agreement regarding treatment, due to a lack of data for the paediatric age group. The group agreed that implementation of adult recommendations for paediatric care might be reasonable, but robust paediatric trials are needed.

## Conclusion

The group made a suggestion for a standard of good clinical practice for RP in children. Our aim is that this will facilitate a large multicentre prospective follow-up study of children with RP.

## Disclosure of interest

None declared.

